# Perception and practices of menstruation restrictions among urban adolescent girls and women in Nepal: a cross-sectional survey

**DOI:** 10.1186/s12978-020-00935-6

**Published:** 2020-06-01

**Authors:** Amrita Mukherjee, Mingma Lama, Uddhav Khakurel, Alok Nath Jha, Fatima Ajose, Sanjeev Acharya, Kristina Tymes-Wilbekin, Marni Sommer, Pauline E. Jolly, Pema Lhaki, Sadeep Shrestha

**Affiliations:** 1 Department of Epidemiology, School of Public Health, University of Alabama at Birmingham, Birmingham, AL USA; 2 Nepal Fertility Care Center (NFCC), Lalitpur, Nepal; 3 University of Michigan, School of Public Health, Ann Arbor, MI USA; 4 Department of Sociology, University of Alabama at Birmingham, Birmingham, AL USA; 5 Columbia University, Mailman School of Public Health, New York, NY USA

**Keywords:** Menstrual perceptions, Menstrual practices, Menstrual restrictions, Women, Adolescent girls, Nepal

## Abstract

**Background:**

Menstruation, a natural biologic process is associated with restrictions and superstitious beliefs in Nepal. However, factual data on women’s perspectives on menstrual practices and restrictions are scarce. This study aimed to assess socio-cultural perceptions of menstrual restrictions among urban Nepalese women in the Kathmandu valley.

**Methods:**

Using a clustered random sampling, 1342 adolescent girls and women of menstruating age (≥15 years) from three urban districts in the Kathmandu valley completed a survey related to menstrual practices and restriction. This was a cross-sectional survey study using a customized program allowing pull-down, multiple choice and open-ended questions in the Nepali language. The self-administered questionnaire consisted of 13 demographic questions and 22 questions related to menstruation, menstrual hygiene, socio-cultural taboos, beliefs and practices. Univariate descriptive statistics were reported. Unadjusted associations of socio-cultural practices with ethnicity, education, four major social classes, three major religions, marital status and family type were assessed using logistic regression models.

**Results:**

More than half (59%) of the participants were aged between 15- < 25 years. The majority were Hindus (84.5%), reported not praying during menstruation (83.1%) and were encouraged by their mothers (72.1%) to practice a range of menstrual restrictions. Purifying either the kitchen, bed, bedsheets or other household things on the fourth day of menstruation was reported by 66.1% of the participants, and 45.4% saw menstruation as a “bother” or “curse.” There were differences among social classes, where participants of the Janajati caste, an indigenous group, were more likely to enter places of worship [OR (95%CI): 1.74 (1.06–2.86)] and pray [OR (95%CI): 1.79 (1.18–2.71)] while menstruating, compared to the Brahmins. Participants with a master’s degree were more likely to pray while menstruating, compared to participants with less than a high school education [OR (95%CI): 2.83 (1.61–4.96)].

**Conclusion:**

This study throws light on existing social discriminations, deep-rooted cultural and religious superstitions among women, and gender inequalities in the urban areas of Kathmandu valley in Nepal. Targeted education and awareness are needed to make changes and balance between cultural and social practices during menstruation.

## Plain English summary

Menstruation is a natural biologic process. However, in many parts of the world societal taboos and stigmas over menstruation still exist. The extreme practice of *Chhaupadi,* a century old Hindu tradition of isolating menstruating women in poorly ventilated menstrual huts is still practiced in certain areas of far western Nepal. Even though the practice of *Chhaupadi* has never been prevalent among urban Nepalese women, because of Nepal government’s initiatives on education, hygiene and socio-cultural awareness, the deeply-rooted cultural and religious belief that menstruation is spiritually polluting still exists in the Nepali society. Consequently, adolescent girls and women of menstruating age are often victims of menstrual restrictions. Avoiding entering the temple, not attending religious or social gatherings, not touching plants or male members of the family, purifying the bed on the fourth day of menstruation, are just some of the common menstrual practices or restrictions followed by menstruating women. However, we do not know much about the perceptions of menstrual practices and restrictions among urban Nepalese women. In this study we surveyed 1342 women aged 15 years or above, from three urban districts in the Kathmandu valley. In the survey, we included questions on basic demographic characteristics, menstrual practices and perceptions of these practices. The majority of the participants were Hindus and reported not praying during menstruation. Nearly two-thirds of the participants were encouraged by their mothers to follow menstrual restrictions. Level of formal school-based education had a positive effect. More educated participants were less likely to follow the restrictions, compared to less educated participants. Menstrual practices and restrictions varied by participants’ social classes; Brahmins were more likely to follow the menstrual restrictions compared to Janajati participants. Understanding awareness and beliefs in the communities will help in exercising the rights and personal freedom with everyday practices during menstruation.

## Introduction

Menstrual practices and beliefs are often constructed from gender, religion and culture [1]. Menstruation is tabooed or stigmatized in many parts of the world, especially in low and middle income countries in Asia and Africa [2]. Instead of being accepted as a natural biological process signifying a girl’s entry into adulthood, for many women and girls, menstruation is associated with restrictions, shame and superstitious beliefs [3]. Menstruating girls and women, specifically in Hindu communities are taught to suffer in silence during menstruation; many restrictions are imposed on them and menstrual hygiene practices are poor [3]. Further, the majority of these women do not have access to proper toilets, clean water, sanitary napkins/ menstrual pads/ tampons or the privacy to change or dispose of menstrual hygiene materials [4]. They are embarrassed to have their periods and rely on used, damp, cotton cloths or rags to control menstrual bleeding [5]. Because of social taboos, women, especially in rural remote areas, are unable to properly wash and dry used menstrual cloths; reusing unhygienic cotton cloths instead of clean sanitary napkins and cloths is a common practice in low and middle income countries [5, 6]. There have been multiple reports of women developing reproductive tract infections (RTIs), abnormal vaginal discharge and other health conditions due to poor menstrual hygiene practices in countries like India, Nepal and Bangladesh [7–11]. However, not much has changed over the years; socio-cultural beliefs regarding menstruation are so deeply ingrained in some societies in Nepal that women believe themselves to be impure, they feel embarrassed of their natural bodily function and are fearful of being blamed/ bringing bad fortune if they do not strictly follow the menstrual restrictions imposed on them by the society [12].

Everyday approximately 237, 250 women menstruate in Nepal; it has been reported that over 89% of women in Nepal experience some form of restriction and exclusion during menstruation [13, 14]. For instance, in remote areas of far and mid-western rural Nepal, women of reproductive age undergo an extreme form of menstrual seclusion practice, ‘*Chhaupadi*’, where they are required to distance themselves from the community during menstruation [3]. *Chhaupadi*, a complex socio-cultural tradition in remote areas of Nepal, is deeply-rooted in religious beliefs and social hierarchies. In a country where more than 80% of the population practices Hinduism, ritualistic purity is a basic tenet [3, 12]. This patriarchal Hindu tradition of *Chhaupadi* considers menstruating women to be impure; imposes multiple socio-cultural restrictions including ostracizing women from their home during menstruation, impeding their access to proper sanitation facilities, menstrual hygiene, healthcare and even clean water [3]. Girls experiencing menstruation for the first time (menarche) are banished from their homes and are required to live in livestock sheds or menstrual huts, also known as ‘*Chhau*’ in local language, for at least 14 days starting with the first day of menarche [3, 12]. Women giving birth are not spared from the ordeal of *Chhaupadi* either, as expecting mothers are isolated in *Chhau* huts during and after childbirth, compromising the wellbeing of both mother and the baby [10]. Despite the Nepal government’s initiative of banning the practice of *Chhaupadi* in 2005, followed by the “*Chhaupadi Pratha”* Elimination Directive in 2008, the practice still exists in some areas and communities in Nepal [15, 16]. Even though this extreme practice has never been prevalent among urban Nepalese women, some socio-cultural restrictions during menstruation still exist in other societies across Nepal, specifically among different castes [17–19]. Some of the common socio-cultural beliefs in various castes in Nepal related to menstruation include: not touching a male member of the family, plant, tree or fruit during menstruation, not consuming pickles or dairy products, eating alone during menstruation, not entering the kitchen or places of worship, not visiting relatives, or not attending social or religious gatherings [12, 20]..

Most existing studies on menstrual practices in Nepal have focused on the practice of *Chhaupadi*. However, even in regions and communities in Nepal where this extreme tradition is not practiced, menstrual taboos still affect women’s daily lives. While rituals are often imposed on menstruating Nepali women, their perceptions are important to assess in order to implement programs targeting changes in behavioural expectations. To date, limited studies have focused on adolescent girls’ and women’s perspectives on existing menstrual practices and restrictions in Nepal [3, 20]. The objective of this study was therefore to assess the socio-cultural perceptions of menstrual restrictions among urban Nepalese adolescent girls and women in the Kathmandu valley.

## Materials and methods

### Study design and data collection

This cross-sectional descriptive study used data collected from surveys conducted between May 15th and August 15th, 2018 in the Kathmandu valley, the most developed and populated place in Nepal. Adolescent girls and young women of menstruating age (15 years and above) from three urban districts in the Kathmandu valley (Bhaktapur, Lalitpur and Kathmandu – the capital city of Nepal) were included in the survey. Twelve clusters were created by selecting four densely populated areas from each of the three districts. Clustered random sampling was used and 1468 women were surveyed face-to-face on menstrual practices and perceptions of these practices. This was a one-time survey, completed in one sitting and no follow-up was involved. The study tool used was a pre-designed, pre-tested, structured and self-administered questionnaire which was developed and translated into the local Nepali language. The participants were informed about the purpose of the study and were briefed about the questionnaire. They were also informed about the confidentiality of the survey, so that they would provide more reliable answers. Signed informed consent was obtained from each of the survey participants and then participants were provided with a tablet to fill in their responses. There was no specific time duration given to answer all the questions. This was because our participants varied in reading ability, and it was important for them to have the time they required to complete the survey. The participants were able to ask for any clarifications with the investigators. For those who were illiterate, the trained surveyors helped with the consent and reading the questions and entering the answers. Consenting participants were invited from the busy street to a study tent where the survey was conducted. No personal identifiers were recorded; anonymity and confidentiality of the study participants were maintained throughout the study period. De-identified data was collected on password protected tablets. The study was approved by the Ethics Review Board (ERB) at the Nepal Health Research Council (NHRC) and by the Institutional Review Board (IRB) at the University of Alabama at Birmingham (UAB).

### Study population

Fourteen hundred and sixty-eight urban Nepalese women aged 15 years and above from 3 districts in the Kathmandu valley were included in the survey. Of 1468 participants, 1342 completed the survey and were included in the analysis. Cut-off age of 15 years and above was used to exclude women who had not started menstruating. Participants were included in the study based on the following inclusion criteria:

- Aged 15 years or above at the time of survey
- Had started menstruating
- Spoke Nepali
- Provided informed consent
- Were of Nepali nationality

### Survey methodology and variables of interest

Survey questionnaires were designed and developed by investigators at the Nepal Fertility Care Center (NFCC) and UAB, based on socio-cultural appropriateness about menstruation, menstrual hygiene and practices in Nepal. The management information system team at NFCC created a customized program similar to ‘Epi Info’ developed by the Centers for Disease Control and Prevention (CDC) [21]. The program allowed pull-down, multiple choice and open-ended questions in the Nepali language. The survey consisted of 13 demographic questions and 22 questions related to menstruation, menstrual hygiene, socio-cultural taboo, beliefs and practices. All female surveyors were centrally trained before the study and were well versed with the content and objectives of the survey. Quality control and assurance were maintained throughout the study period. Survey data were collected on tablets protected with multiple layers of passwords that were changed daily. Literate participants entered their data themselves directly on study tablets and for participants who could not read or write, the data were entered by female study staff. The data were translated into English by investigators who were fluent in both Nepali and English.

Demographic variables included age, religion, ethnicity, marital status, education, employment, housing status/accommodation and information on the participants’ household. Information on participants’ perceptions on menstrual hygiene, prevalent socio-cultural and religious practices/ restrictions during menstruation were recorded using the Likert scale (Strongly agree/ agree/ neither agree nor disagree/ disagree/ strongly disagree). List of the demographic variables and variables/ questions on menstrual hygiene, practice, social and cultural perceptions are provided in Supplementary Document 1, Additional File 1.

### Statistical analysis

Univariate descriptive statistics are reported using frequency (percentages). Both column and row percentages are reported as appropriate. Logistic regression was used to report the unadjusted associations of socio-cultural practices with ethnicity, education, marital status and family type. Odds ratio (OR) and 95% confidence intervals (95% CI) are reported. Statistical significance was set at 0.05 and two-sided *p*-values are reported. Survey data was imported into excel sheets and all statistical analyses were performed in SAS 9.4 (Cary, NC).

## Results

Of 1468 women surveyed, 1342 completed all questionnaires in the survey and were included in the analysis. Table 1 shows the demographic and cultural characteristics of 1342 participants who completed the survey. More than half (59%) of the participants were aged between 15- < 25 years, and only 5.5% were 45 years or above at the time of the survey. The majority of participants were Hindus (84.5%), followed by Buddhists (10.8%). Only 23.6% had a bachelor’s degree and another 5.6% finished master’s level education. Nearly half of the participants (48.4%) worked at government, private or non-government (NGO) organizations. Owning a house (56.4%), living as nuclear families (68.0%), having 4–6 family members (56.9%) were common among the study participants.

**Table 1:** Table showing demographic and cultural characteristics of study participants from three districts in Kathmandu Valley

*Characteristics (N = 1342)*	*Frequency (percentage) [n (%)]*^a^
*Age in years*	
*15 to < 25*	792 (59.0)
*25 to < 35*	304 (22.7)
*35 to < 45*	172 (12.8)
*45 and above*	74 (5.5)
*Religion*	
*Hinduism*	1134 (84.5)
*Buddhism*	145 (10.8)
*Other*	63 (4.7)
*Ethnicity*	
*Brahmin*	269 (20.1)
*Chettri*	290 (21.6)
*Janajati*	678 (50.5)
*Other*	105 (7.8)
*Marital Status*	
*Ever Married*	554 (41.3)
*Never Married*	788 (58.7)
*Highest Education*	
*< High School*	484 (36.1)
*HS/ trade/ vocational degree*	465 (34.7)
*Bachelors*	317 (23.6)
*Masters*	76 (5.6)
*Ever studied outside Nepal*	
*Yes*	64 (4.8)
*No*	1278 (95.2)
*Profession/ employment*	
*Home-maker*	332 (24.7)
*Business*	361 (26.9)
*Govt./ NGO/ private job*	649 (48.4)
*Lived in Kathmandu Valley for*	
*Less than a year*	109 (8.1)
*1–5 years*	213 (15.9)
*6–10 years*	172 (12.8)
*> 10 years*	296 (22.1)
*Whole life*	552 (41.1)
*Housing status*	
*Renter*	585 (43.6)
*Owner*	757 (56.4)
*Family type*	
*Nuclear*	912 (68.0)
*Joint*	430 (32.0)
*Number of members in the household*	
*1–3*	363 (27.0)
*4–6*	763 (56.9)
*7–9*	165 (12.3)
*≥10*	51 (3.8)
*Number of bedrooms*	
*1*	163 (12.1)
*2*	359 (26.8)
*3*	309 (23.0)
*4 or more*	511 (38.2)

Notes: ^a^ = n (%) frequency (column percentage)

Table 2 shows socio-cultural practices among urban Nepalese women in the Kathmandu valley during menstruation. Of all the study participants, 21.1% reported not attending school or work while menstruating and not sleeping in their usual beds while menstruating was reported by 20.6%. Not touching plants/ fruits/ vegetables during menstruation was reported by 47.8% of the participants; 38.4% avoided entering the kitchen and 30.5% avoided eating with family members while menstruating. More than half of the participants (52.1%) reported not mentioning menstruation openly; 41.6 and 39.1% refrained from visiting relatives and attending social gatherings while menstruating, respectively. Nearly two-thirds of the participants (66.1%) reported purifying either the kitchen, bed, bedsheets or other household things on the fourth day of menstruation, with those purifying their beds forming the majority (55%). Only 10.6% of the participants entered places of worship and 12.6% attended religious gatherings while menstruating.

**Table 2:** Table showing socio-cultural practices followed by study participants while menstruating

*Menstrual Practices (N = 1342)*	*Frequency (percentage)* *N (%)*
*Touches plant/ fruits/ vegetables*	700 (52.2)
*Prays*	227 (16.9)
*Attends school/ work*	1059 (78.9)
*Sleeps in her usual bed*	1066 (79.4)
*Touches male members of the family*	901 (67.1)
*Eats with family*	932 (69.5)
*Enters the kitchen*	827 (61.6)
*Cooks food*	730 (54.4)
*Touches water taps*	909 (67.7)
*Touches pickled food (achar)*	695 (51.8)
*Eats dairy products*	907 (67.6)
*Purifies bed and bed sheets on 4th day of menstruation*	888 (66.2)
*Enters places of worship*	142 (10.6)
*Visits relatives*	783 (58.4)
*Attends social gatherings*	817 (60.9)
*Attends religious gatherings*	169 (12.6)
*Wears specific sets of clothes while menstruating*	275 (20.5)
*Mentions menstruation openly*	643 (47.9)
*Person who generally cooks in the family*^*a*^	
*Participant herself*	807 (60.1)
*Participant’s Children*	48 (3.6)
*Participant’s Husband*	43 (3.2)
*Other family members*	543 (40.5)
*Maid*	36 (2.7)
*Others*	61 (4.6)
*Person who cooks in the family while the participant is menstruating*^*a*^	
*Participant herself*	648 (48.3)
*Participant’s Children*	81 (6.0)
*Participant’s Husband*	79 (5.9)
*Other family members*	687 (51.2)
*Maid*	34 (2.5)
*Others*	55 (4.1)
*Things to purify on 4th day of menstruation*^*#*^	
*Kitchen*	245 (18.3)
*Bed*	738 (55.0)
*Whole house*	185 (13.8)
*Nothing*	455 (33.9)

Notes: ^*a*^ = Not mutually exclusive, percentages will not add up to 100

Socio-cultural and religious perceptions measured using the Likert scale are reported in Table 3. When asked about the extreme practice of *Chhaupadi*, more than three-quarters (75.6%) of the participants strongly disagreed or disagreed with the idea that it was okay for women to practice *Chhaupadi*. Another 50.8% strongly disagreed that if a menstruating woman touches a tree/ plant, it will be damaged. Even though 51.6% of the participants agreed that women should not go to places of worship during menstruation, 59.1% agreed and another 22.6% strongly agreed that women should be able to go to wherever they wanted irrespective of their menstrual cycle. Given the opportunity, 50.0% of the participants reported that they would like to stop the practice of not entering the kitchen while menstruating **(**Table 4**)**; 41.4% would like to stop the practice of not going to temple during menstruation. A majority of the participants, heard about menstruation for the first time from their mothers (66.1%), and were encouraged by their mothers (72.1%) to practice menstrual restrictions. Even though 54.6% of the participants accepted menstruation as a blessing, 36.9% saw it as a ‘bother’ and the remaining 8.5% of participants considered menstruation a ‘curse’.

**Table 3:** Perceptions of study participants on socio-cultural practices associated with menstruation, measured using the Likert scale

Perception of practices during Menstruation (N = 1342)	Strongly agree N (%)^a^	Agree N (%)^a^	Strongly disagree N (%)^a^	Disagree N (%)^a^	Neither agree nor disagree N (%)^a^
**Okay for women to practice Chhaupadi**	13 (1.0)	22 (1.6)	693 (51.6)	322 (24.0)	292 (21.8)
**Tree/ plant will spoil if a menstruating woman touches it**	29 (2.2)	93 (6.9)	681 (50.8)	345 (25.7)	194 (14.4)
**Does not matter if a menstruating woman touches a male family member**	343 (25.6)	613 (45.7)	175 (13.0)	160 (11.9)	51 (3.8)
**Important for women to eat separately while menstruating**	18 (1.4)	101 (7.5)	647 (48.2)	525 (39.1)	51 (3.8)
**Better if women do not eat dairy products while menstruating**	25 (1.9)	97 (7.2)	571 (42.5)	546 (40.7)	103 (7.7)
**Important to purify all bed clothes after use on 4th day of menstruation**	103 (7.7)	687 (51.2)	183 (13.6)	202 (15.1)	167 (12.4)
**Good to have specific sets of clothes to wear during menstruation**	44 (3.3)	235 (17.5)	365 (27.2)	530 (39.5)	168 (12.5)
**Good that women do not enter kitchen during menstruation**	28 (2.1)	161 (12.0)	474 (35.3)	577 (43.0)	102 (7.6)
**Women should not go to places of worship while menstruating**	94 (7.0)	692 (51.6)	167 (12.4)	206 (15.4)	183 (13.6)
**It is considerate not to attend religious gatherings while menstruating**	98 (7.3)	552 (41.2)	188 (14.0)	289 (21.5)	215 (16.0)
**A woman should be able to go wherever she wants irrespective of menstrual cycle status**	304 (22.6)	793 (59.1)	92 (6.9)	95 (7.1)	58 (4.3)
**Women should be able to use the washroom in the home when menstruating**	300 (22.4)	687 (51.2)	136 (10.1)	180 (13.4)	39 (2.9)
**When menstruating it is monthly rest/ holiday for women not to go to the kitchen to cook**	137 (10.2)	709 (52.8)	122 (9.1)	215 (16.0)	159 (11.9)
**Nice for women to not have to cook while menstruating**	47 (3.5)	295 (22.0)	230 (17.1)	584 (43.5)	186 (13.9)
**A woman should be able to rest whenever she wants to**	360 (26.8)	781 (58.2)	67 (5.0)	65 (4.8)	69 (5.2)

Notes: n (%)^a^ = Frequency (row percentages)

**Table 4:** Table showing general perceptions and views on menstrual practices among study participants

*Menstruation Practice (N = 1342)*	*Frequency (percentage)*
*First heard about menstruation from*	
*Mother*	887 (66.1)
*Sister*	161 (12.0)
*Aunt*	5 (0.4)
*Grandmother*	83 (6.2)
*Male family member*	2 (0.1)
*Other family members*	23 (1.7)
*Friends*	87 (6.5)
*Teacher*	49 (3.7)
*Books/ internet/ radio/ TV*	32 (2.4)
*Others*	13 (0.9)
*Who encourages the participant to practice menstrual restrictions*	
*Mother*	968 (72.1)
*Sister*	56 (4.2)
*Grandmother*	166 (12.4)
*Aunt*	5 (0.4)
*Husband/ male family members*	6 (0.4)
*Mother-in-law*	13 (0.9)
*Other family members*	52 (3.9)
*Friends*	33 (2.5)
*Teacher*	14 (1.0)
*Other relatives*	29 (2.2)
*Given the opportunity the participant would like to stop the following restrictions*^*#*^	
*Not entering the kitchen*	671 (50.0)
*Not going to temple*	556 (41.4)
*Purifying the house on 4th day of menstruation*	486 (36.2)
*Not touching the plant*	386 (28.8)
*Way the participant sees menstruation*	
*Blessing*	733 (54.6)
*Bothering*	495 (36.9)
*Curse*	114 (8.5)

Notes: ^a^ = Not mutually exclusive, percentages will not add up to 100

Unadjusted associations of some of the socio-cultural practices during menstruation with ethnicity, education, marital status and family type are shown in Table 5. Compared to Brahmins, Janajati participants were more likely to eat with family [OR (95%CI): 3.91 (2.88–5.30)], cook food [OR (95%CI): 4.47 (3.31–6.05)], enter places of worship [OR (95%CI): 1.74 (1.06–2.86)] and pray [OR (95%CI): 1.79 (1.18–2.71)] while menstruating. Janajati and participants of other ethnic groups were less likely to purify their beds on the fourth day of menstruation compared to Brahmin participants [OR (95%CI): 0.59 (0.43–0.81) and 0.43 (0.27–0.68), respectively]. Compared to ever married participants, participants who were never married were 48% less likely to purify their beds on the fourth day of menstruation [OR (95%CI): 0.52 (0.41–0.66)], 2 times as likely to attend school or places or work [OR (95%CI): 2.08 (1.59–2.71)] and enter places of worship [OR (95%CI): 2.07 (1.40–3.05)]. Education level and family type were not found to be significantly associated with the practice of purifying the bed on the fourth day of menstruation (*p*-value 0.45 and 0.85, respectively). Participants with a master’s degree were 2.83 times as likely to pray while menstruating, compared to participants with less than high school education [OR (95%CI): 2.83 (1.61–4.96)]; similarly, participants with a bachelor’s degree were 65% more likely to pray while menstruating compared to those with less than a high school education [OR (95%CI): 1.65 (1.12–2.43)]. Compared to participants who considered menstruation a bother or curse, participants who accepted menstruation as a blessing were 61% more likely to enter places of worship [OR (95%CI): 1.61 (1.12–2.31)], 48% more likely to pray [OR (95%CI): 1.48 (1.11–1.99)] and 75% more likely to attend religious gatherings [OR (95%CI): 1.75 (1.25–2.45)] during menstruation (data not shown).

**Table 5:** Unadjusted associations of socio-cultural practices while menstruating with ethnicity, education, marital status and family type

	Attends school or usual work OR (95% CI)	Eats with family OR (95% CI)	Cooks food OR (95% CI)	Purifies bed OR (95% CI)	Enters places of worship OR (95% CI)	Prays OR (95% CI)
**Ethnicity**						
Brahmin	Reference	Reference	Reference	Reference	Reference	Reference
Chettri	0.97 (0.63–1.49)	1.25 (0.90–1.75)	1.34 (0.94–1.89)	0.79 (0.55–1.15)	1.02 (0.55–1.88)	1.36 (0.84–2.21)
Janajati	0.75 (0.53–1.08)	**3.91 (2.88–5.30)**	**4.47 (3.31–6.05)**	**0.59 (0.43–0.81)**	**1.74 (1.06–2.86)**	**1.79 (1.18–2.71)**
Other	0.71 (0.41–1.23)	**2.99 (1.80–4.98)**	**3.84 (2.39–6.18)**	**0.43 (0.27–0.68)**	1.38 (0.64–2.98)	1.53 (0.82–2.87)
**Marital Status**						
Ever Married	Reference	Reference	Reference	Reference	Reference	Reference
Never Married	**2.08 (1.59–2.71)**	0.81 (0.64–1.03)	**0.75 (0.61–0.94)**	**0.52 (0.41–0.66)**	**2.07 (1.40–3.05)**	**1.85 (1.36–2.52)**
**Highest Education**						
< High School	Reference	Reference	Reference	Reference	Reference	Reference
HS/ trade/ vocational degree	**1.81 (1.33–2.46)**	1.02 (0.77–1.34)	0.88 (0.68–1.14)	0.81 (0.62–1.06)	**1.57 (1.03–2.39)**	**1.51 (1.05–2.16)**
Bachelors	**2.13 (1.49–3.06)**	0.83 (0.61–1.12)	**0.67 (0.50–0.89)**	0.84 (0.62–1.14)	1.26 (0.78–2.03)	**1.65 (1.12–2.43)**
Masters	**2.36 (1.21–4.60)**	0.75 (0.45–1.25)	**0.57 (0.35–0.93)**	0.93 (0.55–1.55)	1.45 (0.68–3.12)	**2.83 (1.61–4.96)**
**Family Type**						
Nuclear	Reference	Reference	Reference	Reference	Reference	Reference
Joint	**0.51 (0.39–0.67)**	**0.65 (0.51–0.83)**	**0.75 (0.60–0.94)**	0.98 (0.77–1.24)	0.88 (0.60–1.29)	0.76 (0.56–1.05)

Notes: *OR (95% CI)* Odds Ratio (95% Confidence Interval); Bold: *p*-value < 0.05

## Discussion

Menstruation related socio-cultural and religious practices are sensitive issues in parts of Nepal and little is known about women’s perspectives on these menstrual practices. This study is one of the first studies to highlight perceptions of urban Nepali women on socio-cultural practices and restrictions surrounding menstruation. Even though the majority of the participants reported attending school or work, social gatherings and eating with family members, less than 20% of the participants attended religious gatherings, entered places of worship or prayed when menstruating. A vast majority still believed in purifying the kitchen, beds or other household materials on the fourth day of menstruation. Women belonging to Janajati and other ethnic groups were less likely to follow the socio-cultural restrictions during menstruation. Our findings highlight deeply-rooted cultural and religious beliefs associated with menstruation in Nepal, and are supported by previous studies that reported resistance to change, entrenched patriarchal values, fear of angering the gods, lack of education on menstrual health, hygiene and societal pressure as some of the major factors contributing to these menstrual restrictions [3, 16, 20].

In the Hindu majority country of Nepal, religion and caste/ethnicity play big roles in sculpting socio-cultural norms. Ritualistic purity is a basic tenet in Hinduism and beliefs that menstruation is unclean are pervasive [20]. While the century old religious tradition of *Chhaupadi* considers menstruating women ‘impure’ and enforces a practice of isolation [12, 20], other practices such as forbidding women from entering the temples and kitchen, cooking food, touching male members of the family, and sleeping on their usual beds when menstruating, which form the very basis of menstrual restrictions at large in Nepal, are more entrenched, widespread and often overlooked. Menstruation-related socio-cultural practices have become significantly less stringent in the past few decades with initiatives from the Nepalese government and other non-governmental organizations (NGOs), specifically those focused on ending *Chhaupadi*; however, many deep-rooted cultural beliefs still exist in the society. Even though women in urban areas of Nepal reported attending social gatherings, visiting workplaces and relatives while menstruating, very few actually reported attending religious gatherings or entering places of worship while menstruating. This selective behaviour among urban Nepalese women could be explained partly by deeply internalized religious beliefs of being ‘impure’ or ‘untouchable’ when menstruating [20, 22]. This selective behaviour could also be partly attributed to the sub-conscious fear of angering the Hindu gods and goddesses by performing/ attending religious rituals, while menstruating.

According to Bista, ‘the absolute belief in *fatalism*: that one has no personal control over one’s life circumstances, which are determined through a divine or powerful external agency’ is deeply engrained in Nepalese psyches [23]. Having its origins in Bahunistic principles, fatalism flourishes in the stratified caste system of Nepal [23]. Since it originated in the Brahmin-Chhetri caste groups, it is possible that women in these higher caste groups may have stronger beliefs in fatalism. Perhaps women consider that it was their fate to have been born as a woman, and thus they must observe the prevailing menstrual restrictions. They cannot change this expectation because it was written into their fate; which in turn keeps them adhering to societal restrictions, without ever questioning them [23]. Not surprisingly, our findings show that socio-cultural and religious restrictions during menstruation were more pervasive among Brahmin women, compared to women from other ethnic groups, and the very origin of fatalism has its roots in the Brahmin group [23].

Overall, a majority of the participants disagreed with the extreme practice of *Chhaupadi*; however, nearly 60% still agreed that purifying beds and bedsheets on the fourth day of menstruation was important. Other socio-cultural restrictions including not eating with family, not touching pickled foods, not cooking food and not visiting relatives when menstruating were not strictly followed by the study participants. Differences in practice of religious and socio-cultural beliefs/ restrictions throw light on the tension between tradition and modernity [20]. The fact that nearly three-quarters of the participants were encouraged by their mothers to practice menstrual restrictions clearly indicates that adherence to menstrual restrictions are instilled and perpetuated by mothers, and interventions that aim to bring social change on this issue should in particular incorporate attention to mothers (or female guardians).

Given that a majority of the participants were young and had never been married, it may be that the sample of urban Nepalese women recruited for this study had more flexible responses towards socio-cultural restrictions associated with menstruation than previous generations. However, when asked about menstrual restriction/traditions that they would like to stop, less than half of the participants reported that they would like to stop not going to temple, not touching plants when menstruating and purifying the house on the fourth day of menstruation. These findings emphasize how patriarchal society in Nepal sets rules and regulations for menstruating women, how women accept and follow the restrictions imposed on them by the society [20, 24]. Another potential explanation as to why these practices perpetuate can be understood through Cockerham’s ‘health lifestyle theory’ [25]. Decision individuals make regarding daily health practices is an outcome of dialectical interplay between social structure (life chances) and agency (life choices). The resulting ‘dispositions to act’ constitute a ‘habitus’ [25]. In simple terms, habitus acts as a ‘cognitive map’ which guides individuals to act in a way, that is deemed appropriate, in a particular social setting [25]. Although around 40% of the participants agreed to stop, perhaps they are simply reproducing the habitus by continuing these practices. They may not feel happy or content towards these practices, however, this is an example of how social structure plays a significant role in shaping and determining routine behaviours including health lifestyle. In this case, perhaps broader social structure (i.e., fatalistic ideology and dominant cultural practices) downplays the role of individual agency (i.e., the desire to stop these practices). Unable to modify habitus, women may feel ambivalent as they keep accepting and practicing menstrual restrictions.

However, there is the possibility that women may want to take a break from these events, as they perform most of these tasks on a daily basis. Even though less than 50% of the participants reported mentioning menstruation openly, the majority saw menstruation as a ‘blessing’ and were less likely to follow religious restrictions. On the other hand, only 8.5% of the participants saw menstruation as a ‘curse’ and nearly one-third of the participants reported not following the tradition of purifying beds, bedsheets or household materials on the fourth day of menstruation; this suggests that the deep-rooted socio-cultural beliefs and superstitions regarding menstruation are fading away with time in urban areas of Nepal. Having acquired higher school-based formal education and not living with conservative joint families had a positive effect against menstruation-related restrictions among urban Nepalese women.

Eradicating poverty and societal inequalities, educating women on menstrual hygiene and health, accessibility and affordability of menstrual hygiene products, access to proper sanitation, clean water and privacy are important steps in menstrual hygiene management (MHM) [3, 26]. Even though our survey did not include information on access to toilets, menstrual hygiene products or their disposal, three-quarters of the participants agreed that menstruating women should be able to use the washroom. Proper sanitation, safe and secure toilets and clean water are not only important for MHM, but are basic human necessities [3, 20]. Social and dietary restrictions should not be allowed to affect the physical and psychological health of menstruating girls and women. The fact that a majority of our study participants did not follow social or dietary restrictions show the positive impact of social workers, public health officials, government and NGO staff in reducing stigma and superstitious beliefs associated with menstruation in Nepal. Even though the menstruation restriction practices are less severe than *Chhaupadi*, educated and urban women are still victims of guilt, insecurity and humiliation. It is also highly likely that menstrual restrictions and perceptions were under-reported by participants in our study and cannot be generalized to all urban women in Nepal. For this study, we did not collect data on menstrual hygiene education and practices, or data from remote, rural areas of Nepal. However, to our knowledge, this is the first major study assessing adolescent girls’ and women’s perspectives on socio-cultural and religious practices and restrictions associated with menstruation in urban districts of Nepal. As such, the questionnaires are not validated; but a pilot study was conducted to assess feasibility and language clarity. This is an understudied field and future efforts will be required to validate the tools in other settings within the Hindu religion framework and culture. Overall, the findings from our study throw light on existing social discriminations, deep-rooted cultural and religious superstitions among women, and gender inequalities in the urban areas of Kathmandu valley in Nepal. While most respondents agree that every menstruating female should have access to all types of facilities and services, the large percentage of women stating their adherence to some form of menstrual restriction exposes a large gap in practice. The need to close this gap between knowledge, attitudes and finally practices among urban Nepali women to bring about social changes is imperative, but impossible without targeted interventions that create environments for women in Nepal to understand and access their right to self-determination.

## Conclusion

Our study findings show that even though most participants disagreed with extreme menstrual restriction practices in the urban areas of Kathmandu valley, Nepal, there were social discriminations, deep-rooted cultural and religious superstitions, and gender inequalities that continue to affect women during menstruation. Targeted education, awareness and interventions with focus on menstrual hygiene and gender sensitization are needed to make practical changes in knowledge, attitude and deep-rooted cultural and religious practices during menstruation.

## Supplementary information

**Additional file 1.** Questionnaire Form.

## Data Availability

The datasets used and/or analysed during the current study are available from the corresponding author on reasonable request.

## Abbreviations

RTI: Reproductive tract infections
ERB: Ethics Review Board
NHRC: Nepal Health Research Council
IRB: Institutional Review Board
UAB: University of Alabama at Birmingham
NFCC: Nepal Fertility Care Center
CDC: Centers for Disease Control and Prevention
OR: Odds ratio
CI: Confidence interval
NGO: Non-government organization
MHM: Menstrual hygiene management
