# Supplementary material for: Perception and practices of menstruation restrictions among urban adolescent girls and women in Nepal: a cross-sectional survey
Source: Reprod Health. 2020 Jun 1;17:81. doi: 10.1186/s12978-020-00935-6 (PMC7268527; doi:10.1186/s12978-020-00935-6)
Supplement: Supplementary file 1 — Additional file 1. Questionnaire Form. [file 12978_2020_935_MOESM1_ESM.docx]

**Perception and Practices of Menstruation Restrictions among Urban Adolescent Girls and Women in Nepal: A Cross-sectional Survey**

***Questionnaire Form***

**DEMOGRAPHIC QUESTIONS**

1. What is your age:

- 15-<20
- 20-<25
- 25-<30
- 30-<35
- 35-<40
- 40-<45
- 45-<50
- 50-<60
- 60-<70
- 70-<75
- 75+

1. What is your religion?

- Hinduism
- Buddhism
- Islam
- Kirat
- Christianity
- Other

1. What is your ethnicity?

- Brahmin
- Chettri
- Janajati
- Other

1. What type of work do you do? (Check all that apply)

- Home
- Business
- Government job
- Private job
- NGO job
- Other job

1. What is your housing status?

- I own my home
- I rent my home

1. How many bedrooms does your home have?

- 1
- 2
- 3
- 4
- 5
- 6

1. How many people live in your home, including you?

- 1-3
- 4-6
- 7-9
- 10 or more than 10

1. What type of family do you have?

- Nuclear
- Joint

1. What is your current or highest level of education? [*can select one option*]

- Primary
- Secondary
- Higher Secondary
- Bachelors
- Masters
- PhD
- Trade/Vocational School
- Other
- No formal schooling

1. Have you ever studied outside of Nepal?

- Yes
- No

1. How long have you lived in Kathmandu?

- Less than a year
- 1-5 years
- 6-10 years
- Greater than 10 years
- Whole life

1. What is your marital status?

- Single
- Married
- Divorced
- Widowed
- Separated

1. Who cooks food in your home?

- Husband
- Children
- Other family member
- Maid
- Own
- Others

**QUESTIONS RELATED TO MENSTRUAL HYGIENE, PRACTICES, SOCIAL AND CULTURAL PERCEPTIONS:**

1. I do the following when menstruating (check all that apply):

- Touch plants, or fruit and vegetable trees
- Pray
- Attend school or work
- Sleep in my usual bed
- Touch male family members
- Eat with my family
- Enter the kitchen
- Touch water taps
- Cook food
- Touch pickled foods (achar)
- Eat dairy products
- Make sure I purify my bedsheets used while menstruating on the 4th day
- Use my usual washroom
- Enter places of worship
- Attend social gatherings
- Attend religious gatherings
- Visit relatives
- Openly mention my period
- Wear a specific set of clothes

1. When menstruating, who cooks for your family? (Check all that apply)

- My husband
- My children
- Another family member
- My maid
- I cook
- Other

1. It is okay for women to practice Chhaupadi

__Strongly Disagree __Disagree __Neither Agree nor Disagree __Agree

__Strongly Agree

1. If the women who is menstruating touches the tree/plant then that will be spoil /die.

__Strongly Disagree __Disagree __Neither Agree nor Disagree __Agree

__Strongly Agree

1. It does not matter is women touch male family members when menstruating

__Strongly Disagree __Disagree __Neither Agree nor Disagree __Agree

__Strongly Agree

1. It is important for women to eat separately from the family when menstruating

__Strongly Disagree __Disagree __Neither Agree nor Disagree __Agree

__Strongly Agree

1. It is better if women do not consume dairy products when menstruating

__Strongly Disagree __Disagree __Neither Agree nor Disagree __Agree

__Strongly Agree

1. If a menstruating woman touches pickled food (achar) it will be contaminated

__Strongly Disagree __Disagree __Neither Agree nor Disagree __Agree

__Strongly Agree

1. It is important that every woman purifies all the bed clothes she used on the fourth day of her menstruation

__Strongly Disagree __Disagree __Neither Agree nor Disagree __Agree

__Strongly Agree

1. What are the things to purify at the 4^th^ day of menstruation (check all that apply):

- Kitchen
- Bed
- Whole house
- Nothing to do

1. It is good to have specific sets of clothes to wear when menstruating (Pant, kurta, sari)

__Strongly Disagree __Disagree __Neither Agree nor Disagree __Agree __Strongly Agree

1. It is a good thing that women do not enter the kitchen when menstruating.

__Strongly Disagree __Disagree __Neither Agree nor Disagree __Agree __Strongly Agree

1. Women should not go to a place of worship when menstruating

__Strongly Disagree __Disagree __Neither Agree nor Disagree __Agree __Strongly Agree

1. When menstruating, it is considerate not to attend religious gatherings.

__Strongly Disagree __Disagree __Neither Agree nor Disagree __Agree __Strongly Agree

1. A woman should be able to go wherever she wants whether she is menstruating or not

__Strongly Disagree __Disagree __Neither Agree nor Disagree __Agree __Strongly Agree

1. Women should be able to use the washroom in the home when menstruating

__Strongly Disagree __Disagree __Neither Agree nor Disagree __Agree __Strongly Agree

1. When menstruating it is monthly rest/ holiday for women not to go to the kitchen to cook.

__Strongly Disagree __Disagree __Neither Agree nor Disagree __Agree __Strongly Agree

1. It is nice for women to not have to cook when menstruating.

__Strongly Disagree __Disagree __Neither Agree nor Disagree __Agree __Strongly Agree

1. A woman should be able to rest whenever she wants to

__Strongly Disagree __Disagree __Neither Agree nor Disagree __Agree __Strongly Agree

1. I first heard about menstruation from (check all that apply):

__My mother __My sister __My aunt __ My grandmother __Male family member __Other family member __My friends __My teacher __ Books, internet, radio, TV __male family member __other

1. I was told about menstrual restrictions by (check all that apply):

__My mother __My sister __My aunt __ My grandmother __Male family member __Other family member __My mother-in-law __Other in-laws __My friends __My teacher __ Books, internet, radio, TV __Other

1. Who encourages you to practice menstrual restriction (check all that apply):

__My mother __My sister(s) __Other family __My husband __My mother in-law __Other in-laws __My friends __My teacher __My religion __My community__Myself __I do not practice menstrual restrictions

1. If you could stop menstrual restrictions (given below), which restriction (s) would you want to stop (check all that apply):

- Not entering the kitchen
- Not going to temple
- Purifying the house in 4^th^ day
- Not touching the plant

1. In which way do you see menstruation

- Blessing
- Bothering
- Curse
